# Influence of Module Design and Concentration Polarization on Pore Size Determination for Nanofiltration Membranes

**DOI:** 10.3390/membranes16020060

**Published:** 2026-02-02

**Authors:** Henrik Schröter, Udo Kragl

**Affiliations:** 1 Institute of Chemistry, University of Rostock, Albert-Einstein-Str. 3a, 18059 Rostock, Germany; henrik.schroeter@uni-rostock.de; 2 Department Life, Light & Matter, Faculty for Interdisciplinary Research, University of Rostock, Albert-Einstein-Str. 25, 18059 Rostock, Germany

**Keywords:** module design, concentration polarization, nanofiltration, Reynolds number, pore size determination, Donnan steric pore model

## Abstract

Nanofiltration is an important part of pressure-driven membrane separation processes. A comprehensive understanding of the interplay between module hydrodynamics, concentration polarization, and solute rejection is essential for predicting NF performance and for scaling up processes. For two different membrane modules, the characterization and determination of concentration polarization as well as pore-size determination according to the Donnan steric pore model are described. The results show that an optimized channel design allows for a more reliable determination of true retention rates without concentration polarization. Differences between observed retention rates and intrinsic retention rates considering mass transfer coefficients can be neglected. These results are obtained at significantly lower cross-flow rates, allowing for better applicability at the lab scale.

## 1. Introduction

Nanofiltration (NF) is an important part of pressure-driven membrane separation processes. NF membranes typically exhibit molecular weight cut-offs between 200 and 1000 g mol^−1^ and are capable of selectively rejecting multivalent ions and moderately sized organic molecules while allowing monovalent salts and water to pass [1,2]. Simply put, the membranes may be described either as porous membranes or as solution-diffusion membranes. At a closer look, the solute retention observed in NF is governed by a combination of mechanisms, including size exclusion, electrostatic (Donnan) interactions, and dielectric effects [3,4].

Beyond intrinsic membrane properties, module design and hydrodynamic conditions strongly influence NF performance. The geometry of the flow channel, spacer configuration, and operating parameters such as cross-flow velocity and transmembrane pressure determine the mass transfer coefficient and, consequently, the extent of concentration polarization—the accumulation of retained solutes near the membrane surface [5,6,7]. This phenomenon reduces the effective driving force for permeation and causes an apparent decline in solute retention relative to the intrinsic membrane selectivity [8]. Accurate determination of retention in NF thus requires distinguishing between inherent membrane characteristics and transport phenomena arising from module design and boundary layer development.

A comprehensive understanding of the interplay between module hydrodynamics, concentration polarization, and solute rejection is essential for predicting NF performance and for scaling up processes involving complex aqueous solutions, such as those encountered in water treatment and product purification applications [9,10,11]. In this context, de Labastida et al. [12] developed a rotating disk-like membrane test cell that yields a spatially homogeneous and quantitatively controllable concentration polarization layer, enabling a concentration polarization-corrected determination of intrinsic ion retentions and their rigorous interpretation by a solution-diffusion electromigration model [12]. In this study, we will present a methodology for how to proceed with a more common layout of membrane modules for investigating concentration polarization and pore size determination.

### 1.1. Concentration Polarization and Mass Transfer

**Figure 1:**
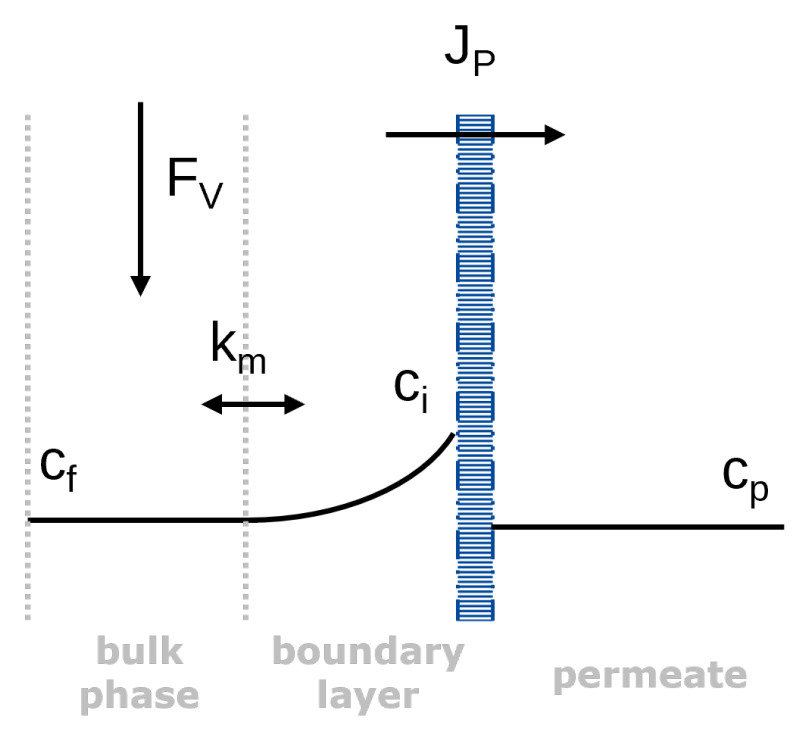
Scheme illustrating the transport through NF membranes according to the solution-diffusion model and film theory.

When a solute is retained by an NF membrane during filtration, this solute is enriched on the membrane surface. Thus, the solute concentration at the membrane–liquid interface $c_{i}$ is increased compared to the bulk concentration of the feed $c_{f}$—a phenomenon termed concentration polarization (Figure 1). This phenomenon has been explained extensively in other studies [13], but essentially, the extent of concentration polarization depends on the exchange rate between the membrane surface and the bulk feed. If this mass transfer is low, it can significantly impact filtration performance and lead to membrane fouling or scaling [10,14]. Even in less extreme cases, concentration polarization reduces the validity of filtration experiments in terms of actually reflecting the performance of the membrane [13].

In many studies, only the observed retention $R_{obs}$ is determined, which is calculated based on the concentration of feed $c_{f}$ and permeate $c_{p}$.

(1) $$\begin{array}{c} R_{obs}=1-\frac{c_{p}}{c_{f}} \end{array}$$

In contrast, the intrinsic retention $R_{i}$ takes concentration polarization into account, as the interface concentration $c_{i}$ is used instead of $c_{f}$. Because $c_{i}$ is the actual concentration that affects the membrane, this approach allows for assessing the separation performance of membranes more accurately.

(2) $$\begin{array}{c} R_{i}=1-\frac{c_{p}}{c_{i}} \end{array}$$

However, $c_{i}$ is experimentally inaccessible and can only be calculated based on models, which require knowledge of the mass transfer coefficient $k_{m}$. This coefficient depends on the fluid dynamics in the membrane cell but also on the diffusion coefficient of the solute of interest (see Section 1.3).

The solution-diffusion model (SDM) assumes that solutes are dissolved in the membrane material upon contact with the membrane surface and are subsequently transported through the membrane due to the difference in chemical potential [15].

This approach can be combined with film theory to yield the following relation [16,17]:

(3) $$\begin{array}{c} \ln\left[\left(1-R_{obs}\right)\;\cdot \;J_{P}/R_{obs}\right]=\ln\left[\frac{DK}{\delta }\right]+\frac{J_{P}}{k_{m}} \end{array}$$

This equation yields a linear relation between $J_{P}$ and $\ln\left[\left(1-R_{obs}\right)\;\cdot \;J_{P}/R_{obs}\right]$, where $\frac{DK}{\delta }$ are solute parameters [16]. The slope of this relation is equal to $\frac{1}{k_{m}}$, allowing for an experimental determination of the mass transfer coefficient based on measurements of the observed retention at different permeate fluxes.

### 1.2. Donnan Steric Pore Model (DSPM)

The Donnan steric pore model (DSPM) and extended models such as the Donnan steric pore model with dielectric exclusion (DSPM-DE) are commonly applied to describe the NF processes of charged and uncharged solutes [18]. The DSPM-DE takes steric exclusion, Donnan exclusion, and dielectric exclusion into account and can thus be used to predict the retention of both charged and uncharged solutes. In the case of uncharged solutes, only the steric term of the DSPM-DE is relevant, as dielectric exclusion and Donnan exclusion do not apply to neutral solutes. As a result, the following relation is obtained [3,19]:

(4) $$\begin{array}{c} R_{i}=1-\frac{K_{i,a}\;\cdot \;\varphi_{S,i}}{1-\left(1-K_{i,a}\varphi_{S,i}\right)\;\cdot \;\exp\left[-\frac{J_{P}L_{e}}{D_{i,\infty }}\frac{K_{i,a}}{K_{i,d}}\right]} \end{array}$$

Here, $L_{e}$ is the effective membrane thickness and the bulk diffusion coefficient is $D_{i,\infty }$. The diffusive hindrance factor $K_{i,d}$, the advective hindrance factor $K_{i,a}$, and the steric exclusion coefficient $\varphi_{S,i}$ are functions of the pore radius $r_{p}$ and the Stokes radius $r_{s}$ of the solute. The ratio of $r_{S}$ and $r_{p}$ is termed the relative solute size λ [20]. For cylindrical pores, the following empirical relations are known to predict $K_{i,a}$ and $K_{i,d}$ based on λ [18,20]:

(5) $$\begin{array}{ccc} K_{i,a}=\frac{1+3.867\;\cdot \;\lambda -1.907\;\cdot \;\lambda^{2}-0.834\;\cdot \;\lambda^{3}}{\left(1+1.867\;\cdot \;\lambda -0.741\;\cdot \;\lambda^{2}\right)} & \; & \mathrm{with}\;\lambda =\frac{r_{s}}{r_{p}} \end{array}$$

(6) $$\begin{array}{c} K_{i,d}=\left\{\begin{array}{cc} \frac{1+\frac{9}{8}\lambda \ln\left(\lambda \right)-1.56\lambda +0.53\lambda^{2}+1.95\lambda^{3}-2.82\lambda^{4}+0.27\lambda^{5}+1.10\lambda^{6}-0.44\lambda^{7}}{{\left(1-\lambda \right)}^{2}} & \mathrm{for}\;\lambda \leq 0.95\\ 0.984\;\cdot \;{\left(\frac{1-\lambda }{\lambda }\right)}^{\frac{5}{2}} & \mathrm{for}\;\lambda >0.95 \end{array}\right. \end{array}$$

(7) $$\begin{array}{c} \varphi_{S,i}=\left\{\begin{array}{cc} 0 & \mathrm{for}\;r_{S}\geq r_{P}\\ {\left(1-r_{S}/r_{P}\right)}^{2} & \mathrm{else} \end{array}\right. \end{array}$$

The filtration of uncharged solutes, such as glucose, to estimate the pore size of NF membranes has been described several times in the literature [8,19,21]. At high permeate fluxes, Equation (4) can be further simplified, as a limiting retention $R_{lim}$ is reached in this case [3]:

(8) $$\begin{array}{c} R_{lim}=1-K_{i,a}\;\cdot \;\varphi_{S,i} \end{array}$$

This equation provides more simple access to the pore radius if the limiting retention is reached experimentally—as expected at high transmembrane pressure—or if it can be predicted accurately from the experimental data.

### 1.3. Theoretical Determination of the Mass Transfer Coefficient

The theoretical mass transfer coefficient is estimated based on calculations of the dimensionless Reynolds (Re), Schmidt (Sc), and Sherwood (Sh) numbers and the empirically determined relations between them.

First, the Reynolds number Re is calculated:

(9) $$\begin{array}{c} Re=\frac{\rho \;\cdot \;v\;\cdot \;d_{h}}{\mu } \end{array}$$

Here, μ and ρ are the viscosity and the density of the solution, respectively. For the diluted glucose solution, the properties of pure water, $\mu_{H_{2}O}=0.891\;\times \;10^{-3}\mathrm{Pa}s$ and $\rho_{H_{2}O}=1000\;kg\;m^{-3}$ are used for the calculations [22]. *v* is the cross-flow velocity, which can be calculated from the volumetric cross-flow rate $F_{V}$ and the channel cross-section *A* or the channel width *a* and channel height *b*, respectively.

(10) $$\begin{array}{c} v=\frac{F_{V}}{A}=\frac{F_{V}}{ab} \end{array}$$

$d_{h}$ is the hydrodynamic diameter, whose calculation depends on the channel geometry. For rectangular channels, $d_{h}$ can be calculated from the channel cross-section *A* and its circumference *U*:

(11) $$\begin{array}{c} d_{h}=\frac{4\;\cdot \;A}{U}=\frac{2\;\cdot \;ab}{a+b} \end{array}$$

In the next step, the Schmidt number is calculated:

(12) $$\begin{array}{c} Sc=\frac{\mu }{\rho \;\cdot \;D_{i,\infty }} \end{array}$$

For glucose, $D_{glucose,\infty }$ = $0.69\times 10^{-9}$ $m^{2}$ $s^{-1}$ was used [23]. The Sherwood number is calculated using the known relation Equation (13), which is applicable for channels or tubes under laminar flow conditions [13].

(13) $$\begin{array}{cc} \; & Sh=a\;\cdot \;Re^{b}\;\cdot \;Sc^{c}\;\cdot \;{\left(\frac{d_{h}}{L}\right)}^{d} \end{array}$$

(14) $$\begin{array}{cc} \; & \;\mathrm{with}\;a=1.62;\;b=0.33;\;c=0.33;\;d=0.33 \end{array}$$

Here, *L* is the length of the channel. For cell II, the projected length of the meandering channel was used. To calculate $k_{m}$, the relation of the Sh and the mass transfer coefficient is applied [22]:

(15) $$\begin{array}{c} Sh=\frac{k_{m}\;\cdot \;d_{h}}{D_{i,\infty }} \end{array}$$

(16) $$\begin{array}{c} k_{m}=\frac{Sh\;\cdot \;D_{i,\infty }}{d_{h}} \end{array}$$

## 2. Materials and Methods

### 2.1. Membranes

For the experiments, the Trisep TS80 NF membrane (Mann+Hummel) was used. Flat membrane sheets were kindly provided by Mann+Hummel, Ludwigsburg, Germany. According to the manufacturer, the membrane is made from polyamide with a non-woven polyester support and exhibits a molecular weight cut-off of less than 300 g mol^−1^. Before the experiments, the appropriate membrane cutouts were placed into ultrapure water for at least 24 h. Afterward, the membranes were placed into the membrane cell and precompacted with ultrapure water at a transmembrane pressure of 20 bar for 5 h.

### 2.2. Filtration Setup

**Figure 2:**
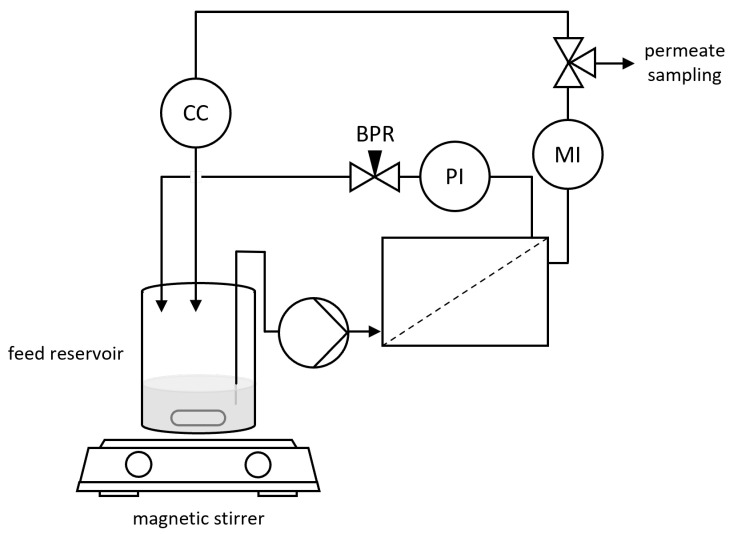
Scheme of the experimental setup. PI—pressure indicator; BPR—back pressure regulator; MI—mass flow meter; CC—conductivity cell.

A bench scale cross-flow NF setup was used for the experiments (Figure 2). The setup consisted of a piston pump (BlueShadow 80P, Knauer, Berlin, Germany) pumping the feed solution over the membrane, a spring-loaded linear back pressure regulator (Ehrfeld Mikrotechnik BTS/Swagelok, Wendelsheim, Germany), and a pressure indicator (Wika M-11, Wika, Klingenberg, Germany, accuracy 0.1 bar). The temperature of the feed reservoir was kept constant by using a heating/stirring plate. A mini CORI-FLOW mass flow meter (Bronkhorst Deutschland Nord, Kamen, Germany) was used to monitor the permeate flow rate. Additionally, the setup was equipped with a flow cell for conductivity measurements, which was not used for the experiments described in this study, however. In order to avoid measurement uncertainties due to mixing effects in the flow cell (V≈0.7mL), permeate samples were taken using a three-way valve before the permeate reached the flow cell.

#### Membrane Cells

**Figure 3:**
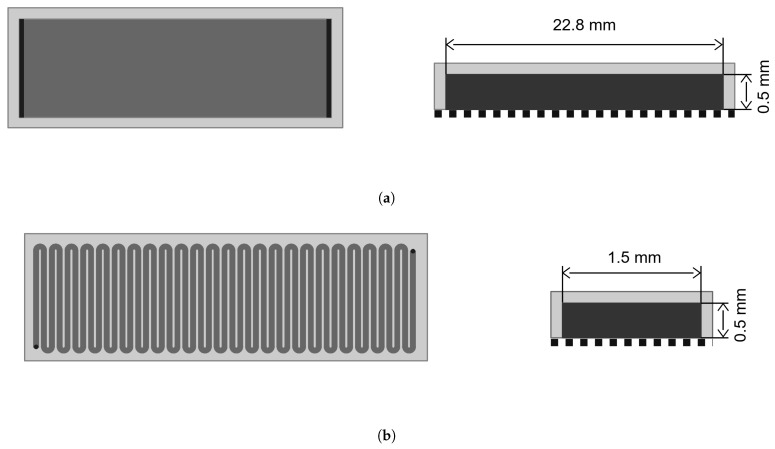
Drawings of the two filtration cells (**a**) I and (**b**) II used for the experiments. Left: top-down view of the channel, where dark gray sections are inlet channels; right: cross-sections of the feed channels. Drawings are not to scale.

Two different membrane cells—denoted cell I and cell II, respectively—were used for the experiments. Both membrane cells were manufactured from stainless steel and sealed with nitrile rubber (NBR) sealing rings. Flat sheet membranes were placed into the membrane cells, and the backside of the membranes was supported by a sintered stainless steel plate. The main difference between the two cells is the design of the feed channel. While cell I has a 2.3 cm wide channel across the whole width of the membrane sheet, the channel for cell II is only 1.5 mm in width and runs in a meandering shape across the membrane surface (Figure 3).

The most relevant properties of the two membrane cells are summarized in Table 1. Cell I had been manufactured multiple years ago and was used for past research projects [24]. As difficulties in the assessment of membrane performance due to increased concentration polarization were observed in more recent projects, the new cell II was manufactured to overcome the limitations of cell I. The design concept of cell II is described in more detail in Section 3.1.

### 2.3. Filtration Procedure

For the filtration of glucose, 0.1 g glucose was dissolved in 500 mL water, yielding the 0.2 g L^−1^ feed solution. This solution was then placed into the feed reservoir of the filtration setup. The feed temperature was set to 25 °C. The pump was started, and the pressure was set using the back pressure valve. The filtration experiments were conducted in recirculation mode, meaning the permeate and retentate were constantly recycled back into the feed reservoir. The permeate flux was monitored throughout the experiment, and the system was equilibrated under each set pressure for at least 30 min. Then, a sample (approximately 1.5 mL) of the feed was taken directly from the reservoir. To obtain a permeate sample, a three-way valve in the permeate outlet was used. To account for the volume of the tubing, 0.5 mL of the permeate was discarded before taking samples of approximately 1.5 mL. Two permeate samples were taken for each set pressure, with a time difference of 10 min in order to ensure stable retention. Filtration experiments with pure water were performed before and after each experiment to assess membrane stability.

The observed retention $R_{obs}$ was calculated based on feed and permeate concentrations. To calculate the permeate flux $J_{P}$ in $L\;m^{-2}\;h^{-1}$ from the flow rate measurements, the permeate flow rate $F_{V,P}$ was divided by the effective membrane area $A_{eff}$—see the Membrane Cells Section for the effective membrane areas of the cells used. Typical volumetric permeate flow rates were 0.73 mL min^−1^ (5 bar, $F_{V}=100\mathrm{mL}\min^{-1}$) for cell I and $0.35\mathrm{mL}\min^{-1}$ (5 bar, $F_{V}=25\mathrm{mL}\min^{-1}$) for cell II.

(17) $$\begin{array}{c} J_{P}=\frac{F_{V,P}}{A_{eff}} \end{array}$$

### 2.4. Chemicals

Unless stated otherwise, ultrapure water was used in all experiments. D(+)-glucose (anhydrous) was purchased from VWR Chemicals (Radnor, PA, USA).

### 2.5. Analytical Methods

The glucose concentrations in the feed and permeate were analyzed using high-performance liquid chromatography (HPLC) on a Knauer, Berlin, Germany HPLC system equipped with a refractive index detector. The measurements were carried out in isocratic mode using a HyperRez XP Carbohydrate H+ column (300 mm × 7.7 mm, 8 μm, Thermo Fisher Scientific, Waltham, MA, USA) equipped with a guard column (50 mm × 7.7 mm, 8 μm, Thermo Fisher Scientific, Waltham, MA, USA). The column temperature was maintained at 60 °C. A 5 mmol L^−1^ sulfuric acid solution was used as the mobile phase at a flow rate of 0.6 mL min^−1^. The injection volume was 20 μL, and the analysis time per run was 15 min. Peak detection and integration were performed using ClarityChrom 8.0 software (Knauer, Berlin, Germany). Prior to analysis, the column was equilibrated (>30 min) with the mobile phase under the same conditions. Glucose was detected at a retention time of 11.5 min and quantified using external standards. Each sample was injected in triplicate to ensure reproducibility.

### 2.6. Calculation of Intrinsic Retentions

The interfacial concentrations $c_{i}$ were estimated using the film theory model (Equation (18)) [21,25], which was analytically solved for $c_{i}$.

(18) $$\begin{array}{cc} \; & \frac{J_{P}}{k_{m}}=\ln\left[\frac{c_{i}-c_{p}}{c_{f}-c_{p}}\right] \end{array}$$

(19) $$\begin{array}{cc} \; & c_{i}=\left(c_{f}-c_{p}\right)\;\cdot \;\exp\left[\frac{J_{P}}{k_{m}}\right]+c_{p} \end{array}$$

The determined $c_{i}$ value was then used to calculate the intrinsic retention according to Equation (2).

### 2.7. Determination of the Pore Size

To determine the pore size, the simplified DSPM for neutral solutes, as formulated in Equation (4), was applied. The procedure was based on Micari et al.’s work [19]. Equations (4)–(8) were implemented in Python 3.13, and a least-squares optimization was conducted to determine the pore radius $r_{p}$ from the glucose retention at different permeate fluxes. Besides $r_{p}$, the effective membrane thickness $L_{e}$ was the second parameter that was optimized. A Stokes radius of 0.365 nm was used for glucose [3]. Details on the fitting procedure and the full implementation of the DSPM for neutral solutes can be found in the Supplementary Materials and the Zenodo repository, respectively.

## 3. Results and Discussion

### 3.1. Rationale for Module Design

**Figure 4:**
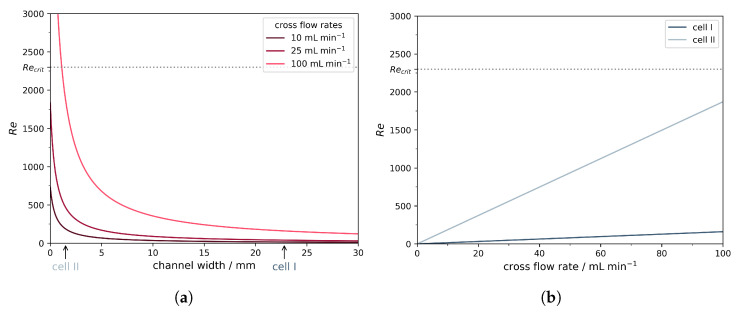
(**a**) Dependency of the Reynolds number on the channel width. A channel height of 0.5 mm was assumed for the calculations. (**b**) Reynolds number depending on the cross-flow rate for the two membrane cells used.

As shown in Figure 4a, the Reynolds number strongly depends on the width of the channel (assuming that the channel height is very small). When the channel width is decreased to about 1 mm, turbulent flow (Re>2300) can be reached even at cross-flow rates as low as 100 mL min^−1^. Because of these calculations, we decided to manufacture a new custom membrane cell with a narrow meandering channel. The channel width of the new cell was 1.5 mm due to manufacturing constraints. However, as Figure 4b illustrates, this design still allows for reaching the upper limit of the laminar flow region with Re=1870 at a flow rate of 100 mL min^−1^, which is easily achievable with most lab-scale piston pumps. In contrast to this, for the original filtration cell I, even a cross-flow rate of 100 mL min^−1^ only yields a Reynolds number of 160. Thus, a major improvement in mass transfer can be expected for cell II while only requiring moderate flow rates.

### 3.2. Experimental Determination of $k_{m}$

The mass transfer coefficient $k_{m}$ was determined experimentally by the filtration of glucose at transmembrane pressures from 1.5 to 20 bar. For membrane cell I, a cross-flow rate of 100 mL min^−1^ was selected, as this was the maximum flow rate possible with the used piston pump. For membrane cell II, a cross-flow rate of 25 mL min^−1^ was selected, as the theoretical considerations predicted an improved mass transfer compared to 100 mL min^−1^ for cell I. The permeate flux was measured throughout the experiment and the glucose retention was determined by HPLC.

**Figure 5:**
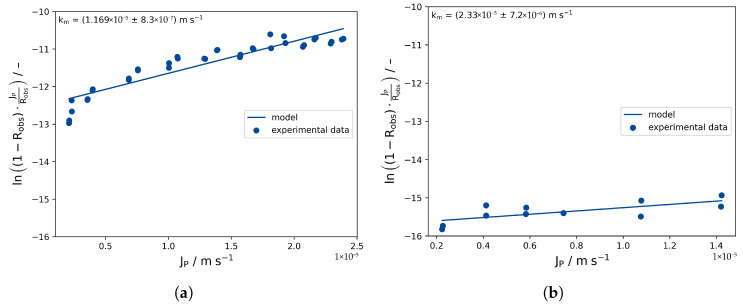
Experimental determination of the mass transfer coefficient based on the solution-diffusion model fo (**a**) cell I and (**b**) cell 2.

Figure 5 shows the resulting data, which were plotted according to Equation (3) to yield $\frac{1}{k_{m}}$ as the slope. Distinct differences can be observed for the two membrane cells. The incline is significantly steeper for membrane cell I, indicating a smaller mass transfer coefficient. This is also reflected by the slopes of the resulting fit. According to the obtained values for $k_{m}$, the mass transfer coefficient is about 1 × 10^−4^ m s^−1^ (≈100%) higher for membrane cell II, while the cross-flow rate is only one quarter of the rate for membrane cell I. However, the uncertainty for the determination of $k_{m}$ from this kind of linearization is quite high for cell II (23% relative error based on the covariance matrix obtained from the fitting algorithm), which may be attributable to the lower sample size and the increase in propagated errors for a lower slope of the fit (see Supplementary Materials).

### 3.3. Comparison to Theoretical $k_{m}$

Theoretical values for $k_{m}$ were calculated based on the methodology described in Section 1.3. As shown in Table 2, theoretical and experimental values for cell I are very similar, indicating that the model describes the mass transfer for straight channels with sufficient accuracy. From an experimental perspective, this may also indicate that an accurate determination of $k_{m}$ is possible via Equation (3).

However, the theoretical mass transfer coefficient for cell II strongly differs from the experimental value and is even lower than the theoretical value for cell I—despite the higher Reynolds number. For cell II, the turns of the meandering channel are not considered in the theoretical calculation of $k_{m}$; instead, the geometry is assumed to be a straight channel of the projected length of the meandering channel. Previous works on mass and heat transfer in meandering and other non-straight channels have shown that mass transfer can be significantly increased through the formation of vortices in the turns [26,27,28], which is not considered by Equation (13). This empirical relation only considers increased mass transfer caused by developing flow in the entrance region through the term $\frac{d_{h}}{L}$. As the channel of cell II is very long in relation to its hydrodynamic radius, the effect of the entrance region on the overall mass transfer of the system is diminished. In contrast to that, the ratio of $d_{h}$ and *L* is larger for cell I, which increases the contribution of the entrance region.

The discrepancy between theoretical and experimental values for $k_{m}$ in the case of cell II shows that a simplified description of cell II as a straight channel is not valid. The higher experimental mass transfer coefficient is likely caused by the meandering design, leading to an increased proportion of developing flow regions. Thus, the overall Sherwood number and subsequently the mass transfer coefficient increase. In a study by Shi et al. [29], the heat transfer coefficient in zigzag-shaped meandering channels increased by factors of two to five when compared to straight channels, which would suggest similar increases for mass transfer. Similarly, for cell II, the discrepancy of theory and experiment suggest that the meandering geometry improves the mass transfer by a factor of two. However, the contribution of the meandering design strongly depends on the channel geometry and requires careful theoretical and experimental assessment for different cell designs and flow conditions [26].

### 3.4. Implications for Modeling

**Figure 6:**
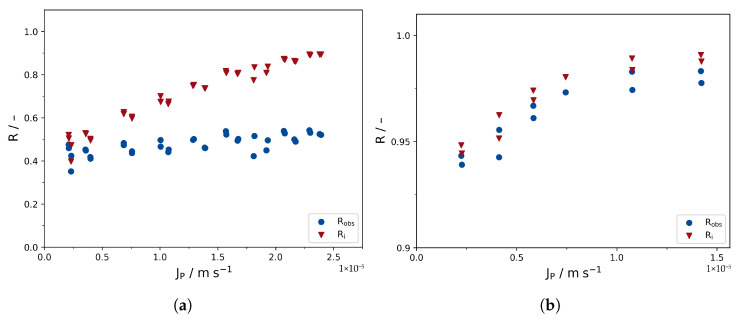
Comparison of intrinsic and observed retentions of (**a**) cell I and (**b**) cell II. The intrinsic retentions were calculated based on the experimental $k_{m}$ values. Note the different y scale for (**b**), as $R_{i}$ and $R_{obs}$ are hardly distinguishable otherwise.

Based on the determined mass transfer coefficients, the observed retentions were converted to the intrinsic retentions via Equations (2) and (18). Figure 6 compares the intrinsic and observed retentions for both membrane cells. The impact of the correction is very strong for cell I—while the observed retentions reach a maximum of 54%, the predicted intrinsic retentions are increased up to 89%. In contrast to that, in the case of cell II, the maximum observed retention is 98%, which is not significantly increased to a maximum intrinsic retention of 99%.

It also becomes obvious that the dependency of the intrinsic retentions on the permeate flux is very different in cell I compared to cell II. While cell I shows a strong increase in intrinsic retention with increasing permeate flux, the intrinsic retention does barely change with permeate flux in the case of cell II. Thus, the limiting rejection seems to have almost been reached at much smaller cross-flow rates. It is also worth noting that the predicted intrinsic retentions for cell I are still below the observed retentions in cell II; thus, they can not reflect the actual performance of the membrane. Logically, the observed retentions in cell II must be the lowest possible values for the intrinsic retentions, as $c_{i}$ cannot reasonably be lower than the bulk feed concentration.

In order to transform the observed retentions from cell I into intrinsic retentions closer to the retentions determined in cell II, $k_{m}$ would have to be significantly lower. Thus, both the theoretical and experimental methodology seem to overestimate $k_{m}$ in terms of its applicability in film theory (Equation (18)), at least in the case of cell II. This illustrates that it is not sufficient to measure or predict $k_{m}$ for a given filtration cell and apply the correction to receive reliable information on the intrinsic membrane performance.

**Figure 7:**
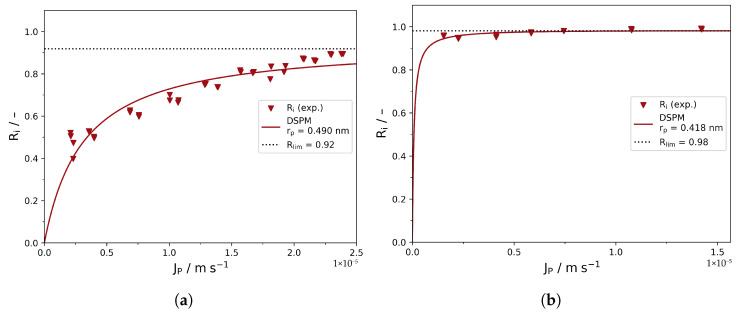
Comparison of the pore radius determination by the DSPM for (**a**) cell I and (**b**) cell II, based on $R_{i}$ values (red data points in Figure 6).

Finally, the DSPM was applied according to Equation (4) to calculate the average membrane pore radius $r_{p}$ based on the intrinsic retention data (Figure 7; red data points from Figure 6 are used). The experiments with cell I and II yield pore radii of 0.49 and 0.42 nm, respectively. Both results are very close to values previously reported in the literature for the TS80 membrane [19].

The limiting retention is predicted to be 92% and 98% for cells I and II, respectively. As is obvious from Figure 6a,b, $R_{lim}$ is an actual extrapolation for cell I, while $R_{lim}$ has already approximately reached the scope of the data points, which is improving the certainty of $R_{lim}$ in the case of cell II.

In an analogy of the arguments presented in the previous paragraphs, the results obtained from cell II are more likely to reflect the true membrane properties as described by the DSPM. As retentions in the range of 98% were actually observed, it seems reasonable that the membrane pore radius is very close to the Stokes radius of glucose ($r_{S}=0.365\;nm$).

It is worth noting that both the observed retentions for cell I and cell II (blue data points in Figure 6) seem to indicate that the system operated close to the limiting retention $R_{lim}$ over the whole pressure range, suggesting the applicability of Equation (8). However, the apparent limiting retention based on the observed retentions seems very different for cell I (approx. 49%) and cell II (approx. 98%), despite the same membrane being used. Based on the limiting retention observed in cell I, the DSPM predicts an average pore radius of 0.97 nm (see Figure S1.7 in the Supplementary Materials), as opposed to the average pore radius of 0.49 nm calculated based on the intrinsic retentions (Figure 6a). In contrast to that, the pore radii predicted by the DSPM from the observed and intrinsic retentions (0.43 nm and 0.42 nm, respectively, see Figure S1.8 in the Supplementary Materials) are almost equal for cell II (Figure 6b). Thus, uncertainties in the determination of $k_{m}$ do not significantly affect the result of the DSPM. However, as the observed retentions are very high anyway in this case, it remains uncertain whether this conclusion remains true for solutes exhibiting moderate retentions as well.

### 3.5. Generalizability

It is important to note that the mass transfer properties and pore radii obtained in this study are specific for glucose and cannot be transferred to arbitrary solutes, i.e., in the DSPM, different uncharged solutes can generally be expected to yield different pore sizes [19,23], while additional separation mechanisms play a role for charged solutes. Similarly, the mass transfer coefficient is specific for the solute, as it correlates with the diffusion coefficient. For solutes with known diffusion coefficients, an estimation of the solute-specific mass transfer coefficient is possible via Equation (3). Hence, for solutes with equal or larger diffusion coefficients than glucose—such as most inorganic ions commonly used in NF [30]—$k_{m}$ will also be similar or larger; thus, negligible concentration polarization can reasonably be assumed for such compounds under the conditions of cell II.

Many novel membrane materials, such as mixed-matrix membranes, rely on adsorption or ion exchange as separation mechanisms [31,32]. Obviously, these mechanisms may significantly change the (apparent) retention of certain solutes independent of the actual pore size while not being considered in the DSPM. Although this increases the uncertainty of the pore size determination, such mechanisms can be excluded by investigating the mass balance during the filtration.

In our opinion, the use of glucose is well-suited as an uncharged model solute, as the Stokes radius and the diffusion coefficient are known, and it is readily available and widely used [8,19,21,23]. By using the procedure described in this study, is it possible to get a general idea of the mass transfer properties of membrane cells. If the mass transfer is adequate—meaning the difference between intrinsic and observed retentions is low—the same experimental data used for the mass transfer investigation may be used to model the pore size of the membrane. Thus, the results obtained from such experiments provide an easy way of assuring sufficient mass transfer and obtaining a descriptor to assess the intrinsic membrane properties (in the form of $r_{p}$) efficiently.

The determination of $r_{p}$ for different membranes provides a means of comparing NF membranes in terms of their pore size—provided the membrane achieves a measurable retention of glucose. In future studies, this procedure should be expanded to other solutes with different Stokes radii and diffusion coefficients, such as glycerol and sucrose [19,33], as this will provide further insights into the robustness of the pore size determination as well as information on the pore size distribution.

## 4. Conclusions

Two lab-scale membrane cells were compared with regard to their mass transfer properties and suitability for model-based membrane characterization. It was shown that the correction of the observed retentions through $k_{m}$ does not result in the same intrinsic retentions for different membrane cells—even though the theoretical and experimental determination of their mass transfer coefficients were in good agreement for cell I. While the qualitative relation between intrinsic retention and permeate flux strongly differs, the average pore radius obtained was in a similar region. Due to the higher observed and intrinsic retentions and improved mass transfer for cell II, the results obtained with this cell are certainly close to the true performance of the NF membrane investigated. In conclusion, membrane cell II seems significantly better-suited for membrane characterization in terms of the intrinsic performance of the membrane. The results highlight the importance of module design and the need for an assessment of concentration polarization—even and especially at the laboratory scale—before any models are applied to the data.

## Figures and Tables

**Table 1:** Properties of the two membrane cells used in this study.

	Membrane Cell I	Membrane Cell II
effective membrane area/${\mathrm{cm}}^{2}$	17.50	13.90
membrane sheet size h × w/cm × cm	8.5 × 3.0	10.7 × 2.8
channel width/mm	22.8	1.50
channel height/mm	0.50	0.50
channel length/mm	77.2	970.2 ^a^

^a^ Projected length of the meandering channel.

**Table 2:** Comparison of experimental and theoretical values for $k_{m}$ for membrane cells I and II.

Membrane Cell	Cross-Flow Rate/mL min^−1^	Exp. $k_{m}$/$10^{-5}$ m s^−1^	Theor. $k_{m}$/$10^{-5}$ m s^−1^
I	100	1.2 ± 0.08	1.4
II	25	2.3 ± 0.72	1.0

## Data Availability

Data supporting this study (raw experimental data, implementations of the models, Python scripts to obtain Figure 5, Figure 6 and Figure 7 from the experimental data, procedures to estimate the errors and robustness of the results, and additional figures) are openly available at Zenodo: https://www.doi.org/10.5281/zenodo.18078087 (accessed on 28 December 2025).

## Supplementary Materials

The following supporting information can be downloaded at https://www.mdpi.com/article/10.3390/membranes16020060/s1, Figure S1.1: HPLC chromatogram of glucose; Figure S1.2: Permeate fluxes for experiments with cell I; Figure S1.3: Permeate fluxes for experiments with cell I (replicate); Figure S1.4: Permeate fluxes for experiments with cell II; Figure S1.5: Sensitivity analysis $k_{m}$ for cell I; Figure S1.6: Sensitivity analysis $k_{m}$ for cell II; Figure S1.7: Pore size determination based on observed retentions (cell I); Figure S1.8: Pore size determination based on observed retentions (cell II); Table S1.1: Parameters and results from the theoretical calculation of $k_{m}$; Table S1.2: Optimization conditions and parameters of the DSPM.

## Abbreviations

The following abbreviations are used in this manuscript:

DSPM	Donnan steric pore model
DSPM-DE	Donnan steric pore model with dielectric exclusion
HPLC	High-performance liquid chromatography
NF	Nanofiltration
SDM	Solution-diffusion model
Mathematical Expressions
*A*	Channel cross-section [m^2^]
$A_{eff}$	Effective membrane area [m^2^]
$d_{h}$	Hydrodynamic radius [m]
$\frac{DK}{\delta }$	Solute transport parameters [−]
$D_{i,\infty }$	Diffusion coefficient (bulk) [m s^−1^]
$F_{V}$	Cross-flow rate [mL min^−1^] or [m^3^ s^−1^]
$F_{V,P}$	Volumetric permeate flow rate [m^3^ s^−1^]
$J_{P}$	Permeate flux [L m^−2^h^−1^] or [m s^−1^]
$K_{i,a}$	Advective hindrance factor [−]
$K_{i,d}$	Diffusive hindrance factor [−]
$k_{m}$	Mass transfer coefficient [m s^−1^]
$L_{e}$	Effective membrane thickness $\left[m\right]$
*L*	Channel length $\left[m\right]$
Re	Reynolds number [−]
$R_{i}$	Intrinsic retention [−]
$R_{lim}$	Limiting retention [−]
$R_{obs}$	Observed retention [−]
$r_{p}$	Average pore radius $\left[m\right]$
$r_{S}$	Solute Stokes radius $\left[m\right]$
Sc	Schmidt number [−]
Sh	Sherwood number [−]
*U*	Circumference $\left[m\right]$
*v*	Cross-flow velocity [m s^−1^]
$\varphi_{S,i}$	Steric exclusion coefficient [−]
ρ	Density [kg m^−3^]
μ	Viscosity $\left[\mathrm{Pa}s\right]$
λ	Relative solute size [−]
