# Supplementary material for: Influence of Module Design and Concentration Polarization on Pore Size Determination for Nanofiltration Membranes
Source: Membranes (Basel). 2026 Feb 2;16(2):60. doi: 10.3390/membranes16020060 (PMC12942572; doi:10.3390/membranes16020060)
Supplement: Supplementary file 1 [file membranes-16-00060-s001.zip › membranes-4098638-supplementary.pdf]

S1

Supporting Information for Article: *Influence of Module Design and Concentration Polarization on Pore Size Determination for Nanofiltration Membranes*

Henrik Schröter[a], Udo Kragl[a,b,*]

[a]Institute of Chemistry, University of Rostock, Albert-Einstein-Str. 3a, 18059 Rostock, Germany

[b]Department Life, Light & Matter, Faculty for Interdisciplinary Research, University of Rostock, Albert-Einstein-Str. 25, 18059 Rostock, Germany

[*]Corresponding author: udo.kragl@uni-rostock.de

List of Figures

S1.1 Glucose HPLC chromatogram 2

S1.2 Permeate fluxes for experiments with cell I 3

S1.3 Permeate fluxes for experiments with cell I (replicate) 3

S1.4 Permeate fluxes for experiments with cell II 4

S1.5 Sensitivity analysis k_m for cell I 6

S1.6 Sensitivity analysis k_m for cell II 6

S1.7 Pore size determination based on observed retentions (cell I) 10

S1.8 Pore size determination based on observed retentions (cell II) 10

List of Tables

S1.1 Parameters and results from the theoretical calculation of k_m 8

S1.2 Optimization conditions and parameters of the DSPM 9

1 Analytical Methods

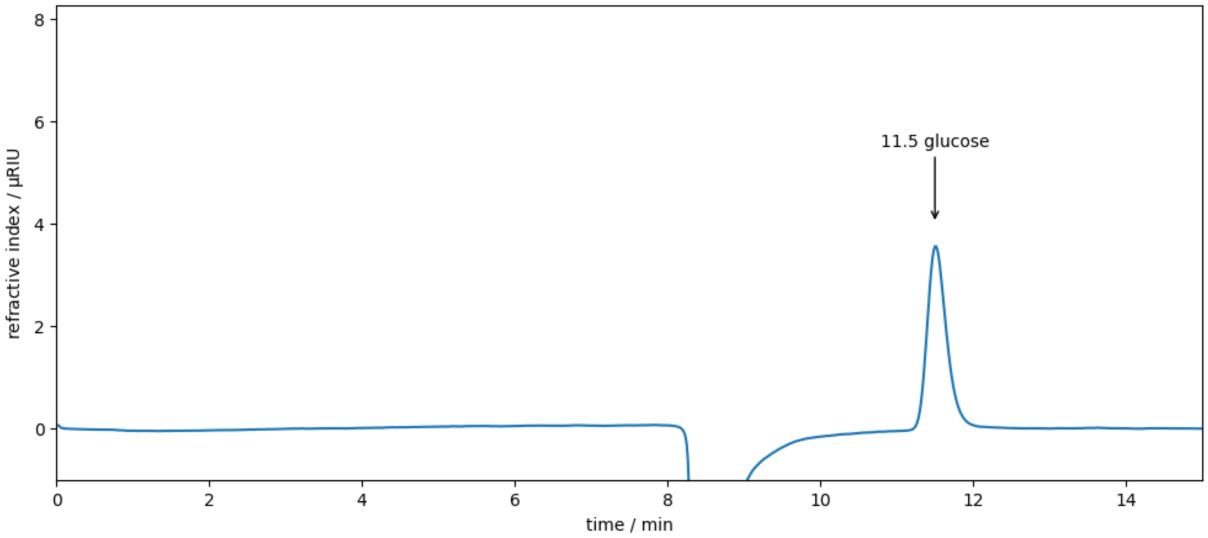

Figure S1.1: Representative HPLC chromatogram for the determination of glucose as used for the retention calculations. Conditions: *HyperRez XP Carbohydrate H+* column (300 mm × 7.7 mm, 8 µm, *ThermoFisher*), equipped with a guard column (50 mm × 7.7 mm, 8 µm, *ThermoFisher*); 60 °C; 5 mmol L^{-1} sulfuric acid (isocratic); 0.6 mL min^{-1}.

2 Filtration Procedure

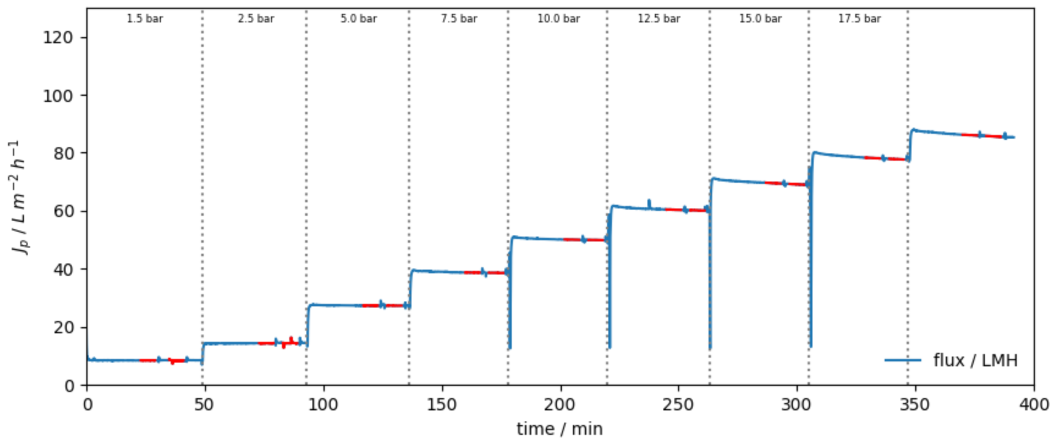

Figure S1.2: Permeate flux measured during the pressure profile during glucose filtration (cell I). Red sections indicate the data points that were averaged to determine the mean permeate flux for the sample which was taken at the end of the red interval.

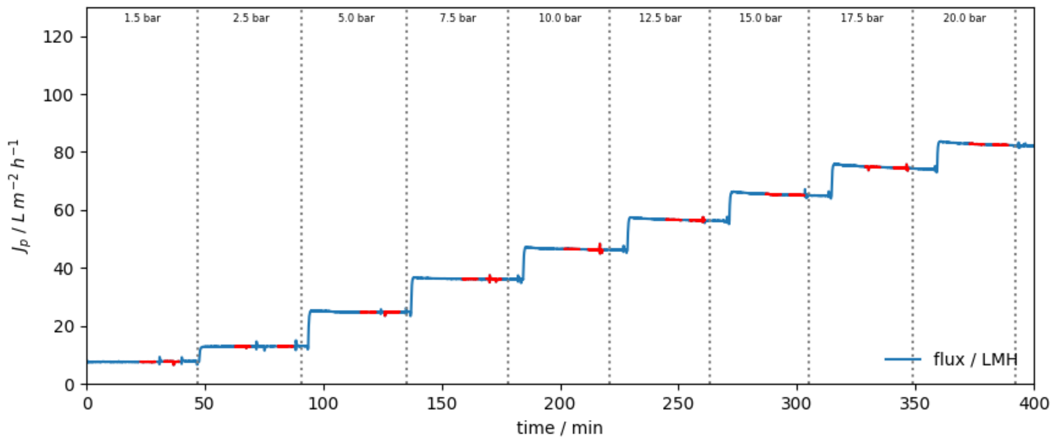

Figure S1.3: Permeate flux measured during the pressure profile during glucose filtration (cell I, replicate of Figure S1.2). Red sections indicate the data points that were averaged to determine the mean permeate flux for the sample which was taken at the end of the red interval.

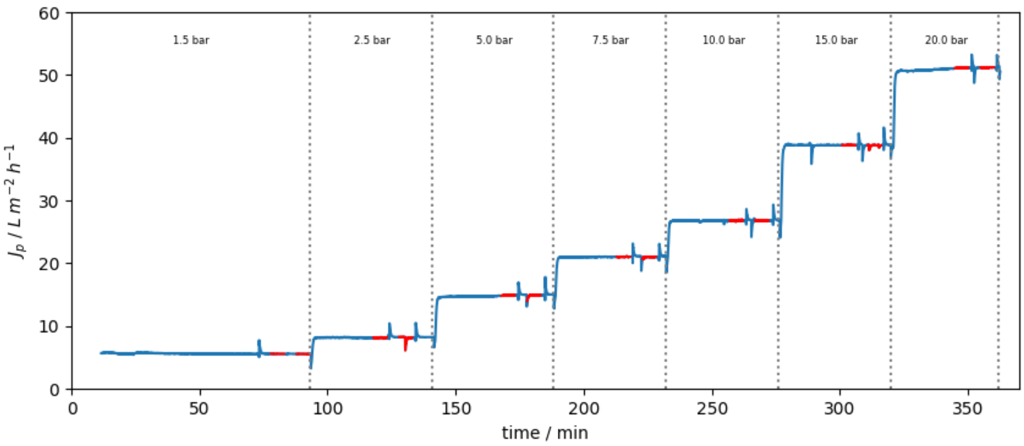

Figure S1.4: Permeate flux measured during the pressure profile during glucose filtration (cell II). Red sections indicate the data points that were averaged to determine the mean permeate flux for the sample which was taken at the end of the red interval.

3 Determination of k_m

3.1 Estimation of the Errors of the Logarithmic Plot

The determination of k_m is based on the following equation:

$$\ln\left[(1 - R_{obs}) \cdot J_P / R_{obs}\right] = \ln\left[\frac{DK}{\delta}\right] + \frac{J_P}{k_m} \tag{1}$$

The error of the left-hand side (LHS) of Equation (1) was estimated based on the Gaussian error propagation:

$$\Delta LHS = \sqrt{\left(\frac{\partial LHS}{\partial J_P} \cdot \Delta J_P\right)^2 + \left(\frac{\partial LHS}{\partial R_{obs}} \cdot \Delta R_{obs}\right)^2} \tag{2}$$

$$= \sqrt{\left(\frac{1}{(R_{obs} - 1) \cdot R_{obs}} \cdot \Delta R_{obs}\right)^2 + \left(\frac{1}{J_P} \cdot \Delta J_P\right)^2} \tag{3}$$

The error for the permeate fluxes J_P was estimated to be around $2 \times 10^{-8}\,\mathrm{m\,s^{-1}}$ based on the standard deviation of the permeate flux data. The error for the observed retention R_{obs} was estimated to be around 0.005 based on the two samples taken under the same conditions.

Based on eq. 3, the error is the highest when J_P and R_{obs} are low. With a permeate flux of $2 \times 10^{-6}\,\mathrm{m\,s^{-1}}$ and an observed retention of 0.35 – the minimal values observed throughout all data used in this paper – the absolute error for the left-hand side term is 0.024, which corresponds to a relative error of 0.1 %. Thus, the error is decreased compared to the relative errors of R_{obs} (1.4 % for $R_{obs} = 0.35$) and J_P (1 % for $J_P = 2 \times 10^{-6}\,\mathrm{m\,s^{-1}}$). However, these considerations are a simplification, as eq. 2 assumes J_P and R_{obs} to be independent variables, which is not the case. Thus, these results are only meant to provide a general idea on the errors for the determination of k_m.

3.2 Estimation of the Standard Error for k_m

$\frac{1}{k_m}$ was obtained as the slope m from the linear curve fit according to Equation (1). The standard error Δm was estimated from the parameter covariance matrix `pcov` – implemented in python as `perr = np.sqrt(np.diag(pcov))`. The standard error for k_m is then defined as:

$$\Delta k_m = \sigma_{k_m} = \left|-\frac{1}{m^2}\right| \cdot \Delta m \qquad \text{with } k_m = \frac{1}{m} \tag{4}$$

3.3 Influence on Intrinsic Retention

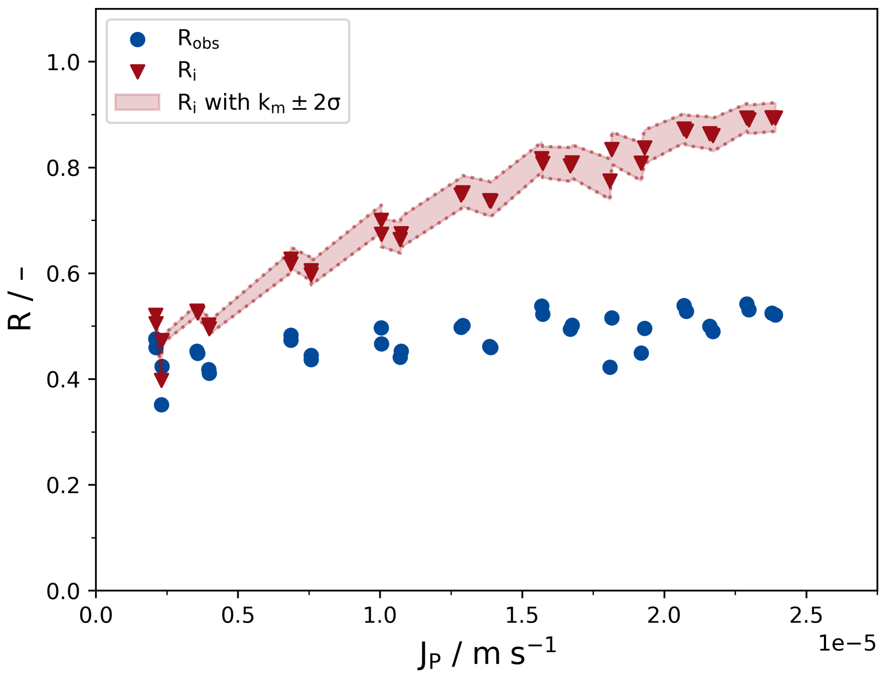

Figure S1.5: Influence of variations of k_m on the calculated intrinsic retentions for cell I. The red area is enclosed by the predicted intrinsic retentions when $k_m + 2\sigma$ and $k_m - 2\sigma$ are used, respectively. The standard deviation σ was estimated based on the parameter covariance resulting from the linear fit applied for the determination of k_m.

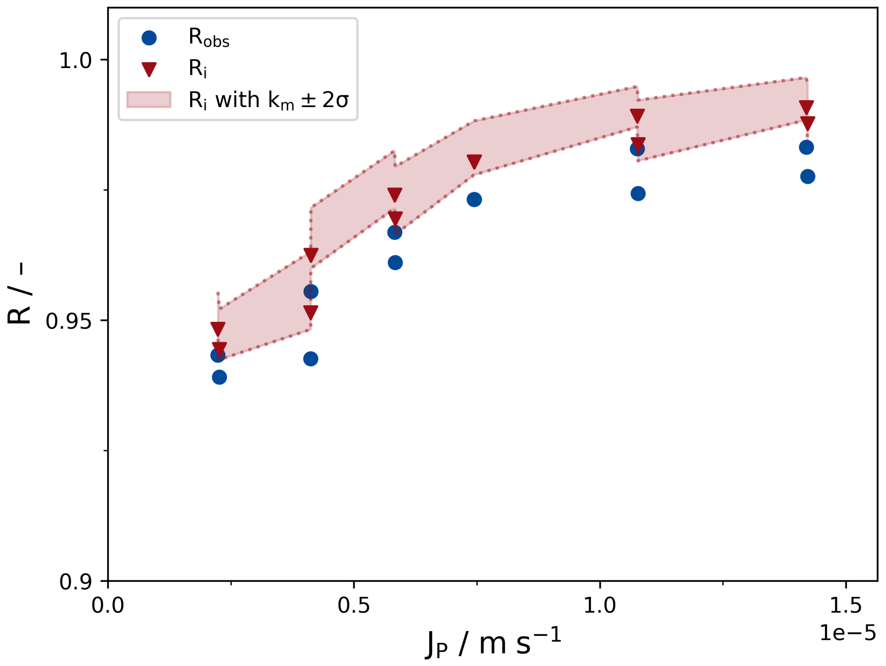

Figure S1.6: Influence of variations of k_m on the calculated intrinsic retentions for cell II. The red area is enclosed by the predicted intrinsic retentions when $k_m + 2\sigma$ and $k_m - 2\sigma$ are used, respectively. The standard deviation σ was estimated based on the parameter covariance resulting from the linear fit applied for the determination of k_m. Note the different y scaling compared to Figure S1.5.

4 Theoretical Determination of k_m

The theoretical determination of k_m was based on the following relations:

$$Sh = a \cdot Re^b \cdot Sc^c \cdot \left(\frac{d_h}{L}\right)^d \tag{5}$$

$$\text{with } a = 1.62;\ b = 0.33;\ c = 0.33;\ d = 0.33 \tag{6}$$

$$k_m = \frac{Sh \cdot D_{i,\infty}}{d_h} \tag{7}$$

The complete procedure is described in the manuscript. Here, it is worth noting that the Sherwood number depends on the length of the channel L according to Equation (5). In the case of cell II (meandering channel), the projected length of the channel was used for L. However, this yielded a drastically lower mass transfer coefficient compared to the experimentally determined value of k_m. This is likely because the turns introduce additional turbulence into the system, enhancing the mass transfer. Regarding Equation (5), the term $\frac{d_h}{L}$ is effectively used to describe the additional turbulence due to developing flow regimes at the entrance. The higher L, the lower is the effect of developing flow regimes in the entrance region on the overall mass transfer behavior. Through the turns of the meandering design, additional sections of developing flow may be introduced throughout the channel. In terms of Equation (5), this effectively reduces the length L appropriate to describe the system.

To investigate the effect of the turns, the calculations of k_m were performed with a value for L which resulted in a k_m value close to the experimentally determined k_m.

The lengths used in these investigations were given as fractions of the total length, divided by the number of meandering turns (49):

$$L = \frac{n}{49} L_{II} \tag{8}$$

By estimating the number of straight channel subunits needed to describe the experimentally determined mass transfer, an idea can be gained of the effect of the turns on the flow conditions. The experimental mass transfer coefficient k_m^{exp} is obtained for n between 4 and 5 (Table S1.1), i.e., in terms of the empirical relation (5), the mass transfer in the module may be described best as a series of about eleven (more precisely 10.9, see third column in Table S1.1) sections with a length of 89.1 mm, for which the developing flow region corresponds to the effects of the meandering turns on the mass transfer.

As the standard deviation of k_m^{exp} was relatively high ($\sigma = 0.72\,\mathrm{m\,s^{-1}}$), the number of subunits required to obtain $k_m^{exp} \pm \sigma$ were also determined (two rightmost columns in Table S1.1).

	cell I	cell II[a]	cell II[b]	cell II[c]	cell II[d]
d_h / mm	0.979			0.750	
L / mm	77.2	970.2	89.1	237.6	39.6
n	–	49	4.5	12	2
F_V / $\mathrm{mL\,min^{-1}}$	100			25	
Re	161			468	
Sc	1485			1485	
Sh	22.8	12.9	28.35	20.51	37.0
k_m / 10^{-5} $\mathrm{m\,s^{-1}}$	1.4	1.0	2.3	1.6	3.0
k_m^{exp} / 10^{-5} $\mathrm{m\,s^{-1}}$	1.2			2.3	

Table S1.1: Parameters and results for the theoretical calculation of k_m based on the module geometry. Different values for L were used in the calculations for cell II to investigate the influence of the meandering design on k_m and compare the theoretical values with the experimental value. Multiples of one straight channel length were thus used. [a]The projected length L_{II} of the meandering channel was used (corresponds to results shown in the manuscript). [b]$L = \frac{4.5}{49} L_{II}$ as to obtain k_m^{exp}. [c]$L = \frac{12}{49} L_{II}$ as to obtain $k_m^{exp} - \sigma$. [d]$L = \frac{2}{49} L_{II}$ as to obtain $k_m^{exp} + \sigma$.

5 Donnan Steric Pore Model

5.1 Optimization Conditions

A least squares fit of the experimental data to the model 9 was performed in python using the library `scipy`. The implementation of the model can be found in the *Zenodo* repository of this publication.

$$R_i = 1 - \frac{K_{i,a} \cdot \varphi_{S,i}}{1 - (1 - K_{i,a}\varphi_{S,i}) \cdot \exp\left[-\frac{J_P L_e}{D_{i,\infty}} \frac{K_{i,a}}{K_{i,d}}\right]} \tag{9}$$

First, the scale of the parameters, the boundary conditions and the tolerances were optimized to yield reliable results for r_p and L_e with varying initial guesses. Based on that, the initial guesses for r_p and L_e were selected (data not shown). Identical parameters were used for all optimizations in this paper (Table S1.2).

Table S1.2: Conditions for the least squares optimization. Parameters are analogous to the parameters used in `scipy.optimize.least_squares`.

**Model Parameters**	
solute	glucose
r_s / nm	0.365
$D_{i,\infty}$ / $m^2\,s^{-1}$	6.9×10^{-10}
**Initial Guess**	
r_p / nm	0.4
L_e / µm	10000
**Boundary Conditions**	
r_p / nm	(0, ∞)
L_e / µm	(0, ∞)
**Optimization Parameters**	
scale r_p	1×10^{-10}
scale L_e	1×10^{-2}
jacobian matrix	2-point
tolerance (`xtol`, `ftol`, `gtol`)	1×10^{-8}
method	`trf`

5.2

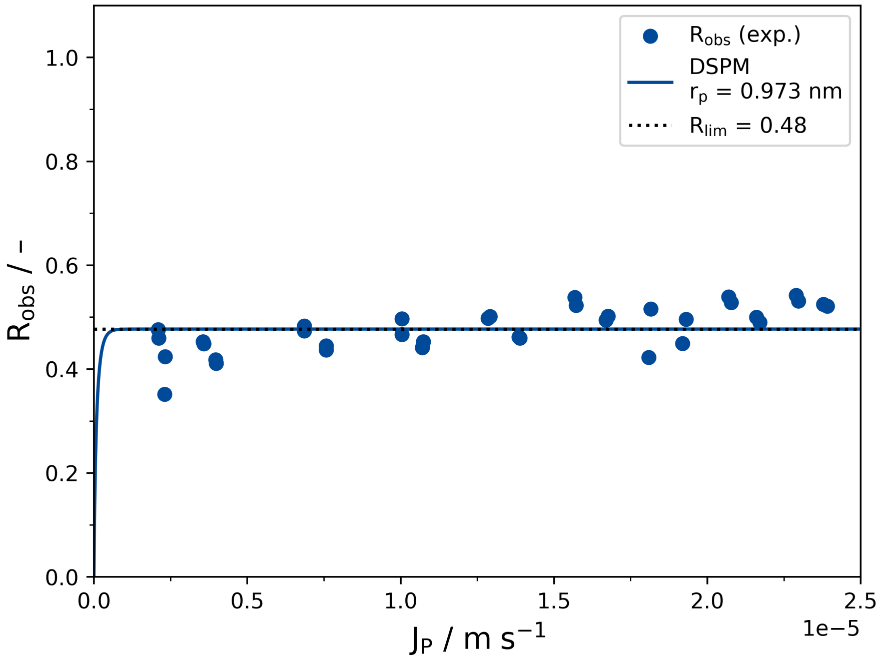

Figure S1.7: Application of the DSPM to the observed retentions determined experimentally for cell I and calculated r_P and R_{lim}. Conditions: Trisep TS80 Membrane, 1.5 to 20 bar, $F_V = 100\,\mathrm{mL\,min^{-1}}$, $0.2\,\mathrm{g\,L^{-1}}$ glucose, 25 °C.

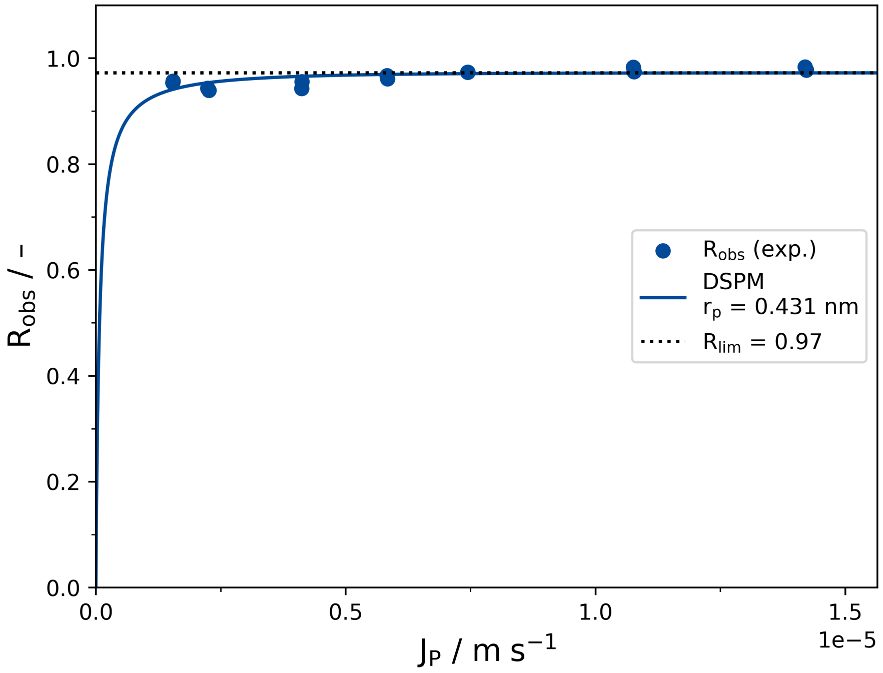

Figure S1.8: Application of the DSPM to the observed retentions determined experimentally for cell II and calculated r_P and R_{lim}. Conditions: Trisep TS80 Membrane, 1.5 to 20 bar, $F_V = 25\,\mathrm{mL\,min^{-1}}$, $0.2\,\mathrm{g\,L^{-1}}$ glucose, 25 °C.
